# Plant–Soil Feedback Effects on Growth, Defense and Susceptibility to a Soil-Borne Disease in a Cut Flower Crop: Species and Functional Group Effects

**DOI:** 10.3389/fpls.2017.02127

**Published:** 2017-12-19

**Authors:** Hai-Kun Ma, Ana Pineda, Andre W. G. van der Wurff, Ciska Raaijmakers, T. M. Bezemer

**Affiliations:** ^1^Department of Terrestrial Ecology, Netherlands Institute of Ecology, Wageningen, Netherlands; ^2^Section Plant Ecology and Phytochemistry, Institute of Biology, Leiden University, Leiden, Netherlands; ^3^Section Green Projects, Delft Research Group, Groen Agro Control, Delft, Netherlands

**Keywords:** chlorogenic acid, chrysanthemum, disease susceptibility, plant–soil feedback, *Pythium ultimum*, plant functional group, phylogenetic distance

## Abstract

Plants can influence the soil they grow in, and via these changes in the soil they can positively or negatively influence other plants that grow later in this soil, a phenomenon called plant–soil feedback. A fascinating possibility is then to apply positive plant–soil feedback effects in sustainable agriculture to promote plant growth and resistance to pathogens. We grew the cut flower chrysanthemum (*Dendranthema X grandiflora*) in sterile soil inoculated with soil collected from a grassland that was subsequently conditioned by 37 plant species of three functional groups (grass, forb, legume), and compared it to growth in 100% sterile soil (control). We tested the performance of chrysanthemum by measuring plant growth, and defense (leaf chlorogenic acid concentration) and susceptibility to the oomycete pathogen *Pythium ultimum*. In presence of *Pythium*, belowground biomass of chrysanthemum declined but aboveground biomass was not affected compared to non-*Pythium* inoculated plants. We observed strong differences among species and among functional groups in their plant–soil feedback effects on chrysanthemum. Soil inocula that were conditioned by grasses produced higher chrysanthemum above- and belowground biomass and less leaf yellowness than inocula conditioned by legumes or forbs. Chrysanthemum had lower root/shoot ratios in response to *Pythium* in soil conditioned by forbs than by grasses. Leaf chlorogenic acid concentrations increased in presence of *Pythium* and correlated positively with chrysanthemum aboveground biomass. Although chlorogenic acid differed between soil inocula, it did not differ between functional groups. There was no relationship between the phylogenetic distance of the conditioning plant species to chrysanthemum and their plant–soil feedback effects on chrysanthemum. Our study provides novel evidence that plant–soil feedback effects can influence crop health, and shows that plant–soil feedbacks, plant disease susceptibility, and plant aboveground defense compounds are tightly linked. Moreover, we highlight the relevance of considering plant–soil feedbacks in sustainable horticulture, and the larger role of grasses compared to legumes or forbs in this.

## Introduction

Plants are the main primary producers in terrestrial ecosystems and as provider of resources, such as litter and root exudates, plants are important determinants of soil biota (Bever et al., 1997; Bardgett and Wardle, 2010). These effects of plants on the soil may differ greatly between plant species as plants vary in the quality and quantity of litter and in the chemical composition of root exudates (Wardle et al., 2003; Bais et al., 2006; Bardgett and Wardle, 2010). Moreover, via their effects on the soil, plants can influence other plants that grow later in the same soil, a phenomenon termed ‘plant–soil feedback’ (van der Putten et al., 2013). Plant–soil feedback effects can be positive, if the succeeding plant grows better in conditioned soil compared to a control soil, and negative, if the growth is reduced (van der Putten et al., 2013). Heterospecific plant–soil feedback (where one species influences the growth of another species) has been recognized as an important mechanism in plant competition and coexistence (Kulmatiski et al., 2008; van der Putten et al., 2013), and there is an increasing interest among ecologists to unravel the mechanisms and determine the generality of plant–soil feedback effects (van der Putten et al., 2013). Although negative conspecific feedbacks are the basis for crop rotation in agriculture, how heterospecific plant–soil feedback influences cultivated plant species is relatively poorly understood as most studies, so far, have focused on interactions among wild plant species (van der Putten et al., 2013; Dias et al., 2015; Detheridge et al., 2016).

Heterospecific plant–soil feedback effects may differ between plant functional groups such as grasses, forbs or leguminous plants (Bezemer et al., 2006; Kos et al., 2015). Legumes, as nitrogen fixers may increase nutrient availability for other plants, and thus may cause positive plant–soil feedback effects (Tilman et al., 1997; Harrison and Bardgett, 2010). Similarly, grasses which have highly branched roots may provide a more suitable habitat for root-associated microbes that have beneficial effects on other plants (Bessler et al., 2009; Pérès et al., 2013; Latz et al., 2015). Clearly, an increase in root surface area that is often found in grasses could also lead to an increase in the abundance of plant antagonists such as root pathogens, but root pathogens of grasses are specialized on monocots, and it is unlikely they will negatively influence plants from another functional group (Cortois et al., 2016) Instead, roots of forb species that typically have higher phosphate contents than grass species are more susceptible to soil-borne pathogens (Laliberté et al., 2015; Zhang et al., 2016). Hence, forbs often host more pathogens than grasses, and are thereby more likely to have a negative feedback effect on later growing plants (Rottstock et al., 2014). As closely related species are more likely to share the same natural enemies and resources (Webb et al., 2006; Gilbert and Webb, 2007), it is legitimate to hypothesize that heterospecific plant–soil feedback effects among closely related species are more negative than among more distantly related species (Brandt et al., 2009; Burns and Strauss, 2011; Anacker et al., 2014; Mehrabi and Tuck, 2015; Münzbergová and Šurinová, 2015).

By growing in the soil, a plant may cause an increase in the density of pathogens in the soil, but at the same time, it may also increase beneficial microbes such as bacteria and fungi that promote plant growth, suppress pathogens or induce resistance in plants against herbivore or pathogen attack (Haas and Défago, 2005; Pineda et al., 2010). Hence, plant–soil feedback effects could influence the susceptibility of a plant to soil pathogens or the disease or pest severity experienced by that plant. We are not aware of any work reporting how plant–soil feedback influences the susceptibility of a plant to soil pathogens, but several studies reported that conditioning of soil by a plant can influence the levels of aboveground herbivory experienced by another plant that grows later in that soil via the feedback effects on the composition and concentration of aboveground secondary compounds of the responding plant (Kostenko et al., 2012; Bezemer et al., 2013; Kos et al., 2015). Soil biota, such as root herbivores, nematodes, and (non-) pathogenic soil microbes can affect plant aboveground primary and secondary compounds (Bezemer et al., 2005; Soler et al., 2012; van de Mortel et al., 2012; Badri et al., 2013), and hence we may expect that plant–soil feedback effects on the susceptibility of a plant to soil diseases will also influence the concentration of aboveground defense compounds in that plant.

In the present study, we examine how plant–soil feedback effects of a wide range of plant species influence the growth and secondary chemistry of the commercial cut flower chrysanthemum and its susceptibility to the soil pathogen *Pythium ultimum*. *Pythium* causes damping off disease to a wide range of plants including chrysanthemum (Weller et al., 2002; Meghvansi and Varma, 2015). Several studies have shown that high abundance and diversity of soil microbes can suppress *P. ultimum* (van Os and van Ginkel, 2001; Yu et al., 2015). We examined in a greenhouse experiment the plant–soil feedback effects of 37 plant species belonging to three plant functional groups on chrysanthemum growth and disease susceptibility. We tested three hypotheses: (i) plant–soil feedbacks will not only influence plant growth, but also influence plant disease susceptibility and plant defense, (ii) soil conditioned by grasses and legumes will positively affect chrysanthemum growth and reduce disease severity relative to soil conditioning by forbs, (iii) species closely related to chrysanthemum will have a more negative effect on chrysanthemum growth than more distantly related species.

## Materials and Methods

### Plant and Pathogen Material

The focal plant in our study is *Dendranthema X grandiflora* (Ramat.) Kitam. cv. Grand Pink [Chrysanthemum, syn. *Chrysanthemum* X *morifolium* (Ramat.) Hemsl., Asteraceae]. Chrysanthemum cuttings were provided by the breeding company FIDES by Dümmen Orange (De Lier, Netherlands). Chrysanthemum is one of the major cut flower crops that is cultivated in soil in greenhouses. In commercial chrysanthemum greenhouses, the soil is disinfected regularly with hot steam to circumvent soil diseases. However, this practice also eliminates the (beneficial) microbial community in the soil and pathogens rapidly recolonize the soil after steaming (Thuerig et al., 2009; Tamm et al., 2010).

The soil–borne oomycete pathogen *Pythium ultimum* (Pythiaceae) was obtained from Wageningen UR Greenhouse Horticulture (Wageningen UR, Greenhouse Horticulture, Bleiswijk, Netherlands). *Pythium ultimum* was isolated from diseased chrysanthemum plants, and cultured on liquid V8 medium (200 ml of organic tomato suspension without added salt, 2 g CaCO_3_, and 800 ml water) at room temperature for 2 weeks. Then, the *P. ultimum* culture was blended in a mixer and filtered to obtain a solution with only oospores based on a modified protocol of van der Gaag and Wever (2005). The oospores concentration was determined by counting (Fuchs-Rosenthal chamber) the oospore number in 1 ml liquid suspensions under the microscope.

### Experimental Set-Up

The experiment consisted of two phases. In the first phase, the conditioning phase, we used 37 plant species to condition soil by growing them in monocultures. In the second phase, the test phase, we measured the effects of the species-specific conditioned soils as inocula on the performance of chrysanthemum plants with and without *P. ultimum* addition.

#### Phase I: Conditioning phase

For the conditioning phase, 300 Kg soil was collected (5–20 cm deep) in November 2014 from a semi-natural grassland that was previously used to grow maize and where agricultural activities ceased in 1995 (Mossel, Ede, Netherlands). The collected soil was homogenized and sieved (1 cm mesh size) to remove coarse fragments and all macro-arthropods. Pots (13 cm × 13 cm × 13 cm) were filled with a homogenized mixture of field soil and sterilized field soil in a 1:1 ratio (total 1.6 Kg soil per pot). Part of the soil was sterilized by gamma irradiation (>25 K Gray gamma irradiation, Isotron, Ede, Netherlands).

Thirty-seven plant species were selected to create conditioned soils (**Table 1**). The species were classified as grasses (9 species), forbs (21 species), or legumes (7 species) (**Table 1**). Most species were wild species that are typical of natural grasslands in Netherlands. *Tagetes minuta* is a domesticated species that was included because of its known disease suppressive properties (Hooks et al., 2010). Seeds of the wild species were obtained from a wild plant seed supplier (Cruydt-Hoeck, Assen, Netherlands) and *Tagetes minuta* seeds were obtained from a garden plant seed supplier (Vreeken seeds, Dordrecht, Netherlands). Seeds were surface sterilized in 3% sodium hypochlorite solution for 1 min, rinsed and germinated on sterile glass beads in a climate chamber at 20°C (16 h/8 h, light/dark).

**Table 1:** List of plant species used in the conditioning phase, their abbreviation used in the manuscript, family and functional group are also presented.

Species	Abbreviation	Family	Functional group
*Agrostis capillaris*	AC	Poaceae	Grass
*Agrostis stolonifera*	AS	Poaceae	Grass
*Anthoxanthum odoratum*	AO	Poaceae	Grass
*Bromus hordeaceus*	BH	Poaceae	Grass
*Festuca filiformis*	FF	Poaceae	Grass
*Festuca rubra*	FR	Poaceae	Grass
*Holcus lanatus*	HL	Poaceae	Grass
*Lolium perenne*	LP	Poaceae	Grass
*Phleum pratense*	PP	Poaceae	Grass
*Carum carvi*	CAC	Apiaceae	Forb
*Achillea millefolium*	ACM	Asteraceae	Forb
*Arnica montana*	ARM	Asteraceae	Forb
*Centaurea jacea*	CJ	Asteraceae	Forb
*Crepis capillaris*	CRC	Asteraceae	Forb
*Hypochaeris radicata*	HR	Asteraceae	Forb
*Jacobaea vulgaris*	JV	Asteraceae	Forb
*Leucanthemum vulgare*	LV	Asteraceae	Forb
*Matricaria recutita*	MR	Asteraceae	Forb
*Tagetes minuta*	TM	Asteraceae	Forb
*Tanacetum vulgare*	TV	Asteraceae	Forb
*Taraxacum officinale*	TO	Asteraceae	Forb
*Arabidopsis thaliana*	AT	Brassicaceae	Forb
*Capsella bursa-pastoris*	CB	Brassicaceae	Forb
*Campanula rotundifolia*	CR	Campanulaceae	Forb
*Hypericum perforatum*	HP	Hypericaceae	Forb
*Prunella vulgaris*	PV	Lamiaceae	Forb
*Thymus pulegioides*	THP	Lamiaceae	Forb
*Plantago lanceolata*	PL	Plantaginaceae	Forb
*Rumex acetosella*	RA	Polygonaceae	Forb
*Galium verum*	GV	Rubiaceae	Forb
*Lotus corniculatus*	LC	Fabaceae	Legume
*Medicago sativa*	MS	Fabaceae	Legume
*Trifolium arvense*	TA	Fabaceae	Legume
*Trifolium pratense*	TRP	Fabaceae	Legume
*Trifolium repens*	TR	Fabaceae	Legume
*Vicia cracca*	VC	Fabaceae	Legume
*Vicia sativa*	VS	Fabaceae	Legume

Five 1-week-old seedlings were transplanted in monocultures in each pot (13 cm × 13 cm × 13 cm), with five replicate pots for each species. A set of five pots filled with field soil (without plants) was also kept in the greenhouse, and served as the “no plant” control for the test phase. In total, the conditioning phase comprised of 190 pots (monocultures of 37 plant species × 5 replicates + no plant pots × 5 replicates). The replicate pots of each species in the conditioning phase were kept separately throughout the experiment. Seedlings that died during the first week of the experiment were replaced. A few seedlings died after transplantation. Therefore, 2 week later, the number of seedlings in each pot was reduced to four. All pots were placed randomly in a greenhouse with 70% RH, 16 h 21° (day) and 8 h 16° (night). Natural daylight was supplemented by 400 W metal halide lamps (225 μmol s^-1^ m^-2^ photosynthetically active radiation, one lamp per 1.5 m^2^). The pots were watered regularly. Ten weeks after transplanting, plants were clipped and the largest roots were removed from the soil as they may act as a source for re-growing plants. Finer roots were left in the soil as the rhizosphere may include a major part of the microbial rhizosphere community. The soil from each pot was homogenized and stored in a plastic bag at 4°C (1 bag for each pot) until used in the test phase. These soils are called “soil inocula” hereafter.

#### Phase II: Test phase

For the test phase, 1 L pots (11 cm × 11 cm × 12 cm; length × wide × height) were filled with a homogenized mixture of 10% soil inoculum (plant species-specific conditioned soil) and 90% sterile soil (see above). Two controls were included in the test phase: 100% sterile soil and 90% sterile soil mixed with 10% field soil that was kept without plants in the greenhouse during the conditioning phase (“no plant” inoculum). Two chrysanthemum cuttings (without roots) were planted in each pot as preliminary work showed that not all cuttings establish properly with this method. Prior to planting, the soil in each pot was well watered and 100 ml half-strength Hoagland nutrient solution was added. The pots were placed on trolleys, each trolley had 48 pots and was tightly covered with a thin transparent plastic foil for 10 days to create a closed environment with high humidity that favors rooting. After 10 days, one of the chrysanthemum cuttings was removed from each pot. Plants were fertilized following grower’s practice: half-strength Hoagland nutrient solution for the first 2 weeks, and single strength Hoagland solution during the following 2 weeks. For the last 2 weeks, the strength was increased to 1.6 mS/cm EC (electrical conductivity). The density of pots on each trolley was reduced 2 weeks after the start of the second phase to 32 pots per trolley so that there was 10 cm space between each pot.

Five days after the transparent plastic foil had been removed, 3 ml of the oospore suspension (ca. 355000 oospores of *P. ultimum*) was added onto the soil next to the stem of each plant allocated to the disease treatment. For plants in the control treatment (non-*Pythium* inoculated), 3 ml water was added. In both treatments, there were two replicate pots for each soil from the conditioning phase. Hence, the feedback phase comprised of 780 pots [(37 plant specific soil inocula + no plant soil inoculum) × 2 disease treatments × 5 soil replicates × 2 replicate pots + 100% sterile soil × 2 disease treatments × 10 replicates]. All pots were randomly arranged in a greenhouse compartment and kept under the same conditions as described for the conditioning phase.

### Plant Performance and Disease Susceptibility

Six weeks after disease inoculation, all plants were harvested. For each plant, the total number of leaves and the number of yellow leaves was recorded and plant yellowness was calculated as the proportion of yellow leaves. The third fully expanded leaf from the top of each plant was then clipped and stored at -80°C for chlorogenic acid analysis (see below). Plants were then clipped at soil level and roots were rinsed from the soil. Shoot and root biomass were oven-dried (60°C for 3 days) and weighed and the root/shoot ratio was calculated. The main symptom of *Pythium* infection is the reduced root system caused by root rot (Agrios, 2005), and thus plant root/shoot ratio is used as an indicator of plant susceptibility to *Pythium*.

### Analysis of Chlorogenic Acid

Chlorogenic acid acts as an important resistance factor in chrysanthemum against plant attackers such as herbivorous insects (Leiss et al., 2009). Chemical analysis was performed using high performance liquid chromatography (HPLC) with UV diode array detection following the procedure outlined by Olszewska (2007). Leaves were freeze-dried and finely ground. Ten mg of ground leaf material was then used for chemical analysis. Each leaf sample was extracted twice. In the first extraction, 1 ml 70% MeOH was added to each sample, vortexed for 0.5 min, then ultrasonicated for 30 min at 20°C, centrifuged for 10 min at 10000 rpm, and labeled. The extraction was repeated so that each sample was extracted by 2 ml 70% MeOH. The extraction was filtered using a 0.2 μm PTFE syringe filter and stored at -20°C until analysis. A standard solution that contained 10 mg chlorogenic acid per 10 ml 70% MeOH was used to produce an external standard curve. In each sample chlorogenic acid was then quantified based on the standard curve. The concentration of chlorogenic acid was determined, and expressed per g leaf dry weight.

### Phylogenetic Analysis

We constructed a phylogenetic tree of the 37 plant species, and chrysanthemum using the program Phylomatic (Webb and Donoghue, 2005), in which a taxon list is matched against a backbone ‘metatree,’ returning a pruned tree of genus-level relationships. The backbone tree is based on the recent phylogenetic hypothesis of the Angiosperm Phylogeny Group (R20120829 for plants). We used the BLADJ algorithm of the Phylocom version 4.1 software package (Webb et al., 2008) to get branch lengths scaled to time, based on clade ages according to Wikström et al. (2001).

### Statistical Analysis

Prior to analyses, data from the two pots with the same soil inoculum replicate of the same disease treatment were averaged. Sterile soil came from the same homogenized source, and therefore these ten replicate pots were kept as 10 replicates. Before conducting analysis, data were checked for homogeneity of variance and normality was confirmed by inspection of the residuals. The overall effects of plant species-specific inocula and pathogen inoculation on chrysanthemum were analyzed using a linear mixed model. In the model, plant species-specific inocula and disease treatment were set as fixed factors, and soil replicate was set as random factor. In this analysis, sterile soil and no plant soil inocula were not included, as they are not species-specific soil inocula.

The pathogen effect was calculated for each soil replicate (including sterile soil and the no plant soil inoculum) as biomass in disease soil minus biomass in no disease soil. One-way ANOVA was used to determine the difference of pathogen effects between soils. A one sample *t*-test was then used to determine for each soil inoculum if the pathogen effect was significantly different from zero. The soil effects (including sterile soil and no plant soil) in the control treatment were compared using one-way ANOVA. *Post hoc* Dunnett tests were performed to compare each plant species-specific inoculum with sterile soil and with the no plant soil inoculum. The analyses described above were done for chrysanthemum aboveground biomass, belowground biomass, leaf chlorogenic acid and root/shoot ratio (Supplementary Figure 1). Plant proportional yellowness was not normally distributed, and thus the analyses were done slightly different. The effects of plant species-specific inocula and pathogen inoculation on chrysanthemum yellowness were analyzed using a generalized linear mixed model with binomial distribution and logit link function, with plant species-specific inocula and pathogen inoculation set as fixed factors, and soil replicate as random factor. The pathogen effect was calculated for each soil replicate (including sterile soil and no plant soil inocula) as proportion yellowness in disease soil minus that in no disease soil. One-way ANOVA was used to determine the difference of pathogen effects between soils. A one sample *t*-test was then used to determine for each soil inoculum if the pathogen effect was significantly different from zero. The soil effects (including sterile soil and no plant soil inoculum) in the control treatment were compared using a generalized linear model. *Post hoc* Dunnett tests were performed to compare each plant species-specific inoculum with sterile soil and with the no plant soil inoculum. To quantify plant–soil feedback effects of a conditioning species on chrysanthemum, the plant–soil feedback effect was calculated as natural log of the (chrysanthemum biomass (aboveground biomass + belowground biomass) on soil conditioned by that species minus average chrysanthemum biomass on sterile soil or no plant inoculum). This calculation was done for both the control treatment and the pathogen treatment. Two-way ANOVA was used to determine the overall effects of conditioning species and disease treatment on plant–soil feedback effects. A one sample *t*-test was used to determine for each species inoculum, if the effect was significantly different from zero.

To compare functional groups of the conditioning plant species (grass, forb, or legume), linear mixed models were used with plant functional group and pathogen inoculation as fixed factors, and soil replicate nested in plant species identity as a random factor, so that each conditioning species was considered a replicate. In this analysis, the sterile soil and no plant soil inoculum were not included, as these treatments were not allocated to a specific plant functional group. *Post hoc* tests were conducted with the functions ‘glht’ (multcomp package) and ‘lsm’ (lsmean package) to assess pairwise comparisons between plant functional groups. The analyses described above were done for chrysanthemum aboveground biomass, belowground biomass, root/shoot ratio and leaf chlorogenic acid. For plant yellowness, a generalized linear mixed model was used (binomial distribution and logit link function), with plant functional group and pathogen inoculation as fixed factors, and soil replicate nested in plant species identity as random factor. The same *post hoc* tests were done for pairwise comparisons of different plant functional groups.

Linear regression analysis was used to test the relationship between the phylogenetic distance of the conditioning plant species to chrysanthemum, and chrysanthemum biomass (aboveground biomass + belowground biomass). Linear regression analysis was also used to determine the relationship between chrysanthemum leaf chlorogenic acid and chrysanthemum aboveground biomass for the control and disease treatment separately. All analyses were performed in R (version 3.0.1, R Development Core Team, 2013).

## Results

**FIGURE 1:**
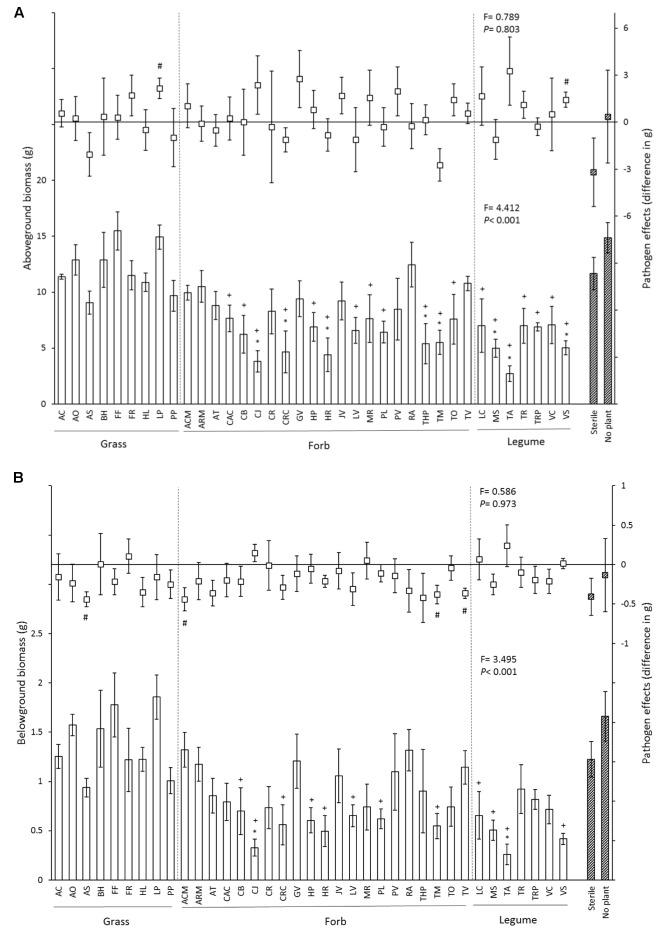
Effects of 37 species-specific soil inocula, no plant inoculum and sterile soil on chrysanthemum aboveground biomass **(A)** and belowground biomass **(B)**. In each figure, bars represent chrysanthemum biomass (mean ± SE) of soil inocula in control soil, and squares represent the pathogen effect on plant biomass (biomass in *P. ultimum* soil – biomass in non-*Pythium* inoculated soil). Striped bars indicate controls. “^∗^” Represents significant difference from the sterile soil (*P* < 0.05). “+” Represents significant difference from the no plant soil inoculum (*P* < 0.05), “#” represents significantly different from zero (*P* < 0.05). Dashed lines separate soil inocula into different functional groups. Species abbreviations are given in **Table 1**. Statistics presented in the lower part of each panel represent the effects of soil on chrysanthemum biomass in control soil, and statistics presented in the upper part of each panel indicate the effects of soil inocula on the disease severity of chrysanthemum biomass.

Above- and belowground biomass of chrysanthemum plants differed significantly between inocula and average root and shoot biomass varied more than threefold (**Figure 1** and **Table 2**). In the control treatment, aboveground biomass of chrysanthemum grown with soil inocula from 8 species (*Thymus pulegioides, Crepis capillaris, Tagetes minuta, Hypochaeris radicata, Centaurea jacea, Medicago sativa, Vicia Sativa*, and *Trifolium arvense*) was significant lower than that of chrysanthemum grown in sterile soil. Compared to the no plant inoculum this was observed for 19 of the 37 species-specific soil inocula (**Figure 1A**). Overall, pathogen addition did not significantly influence plant aboveground biomass, and did not modify the effects of the different soil inocula on chrysanthemum aboveground biomass (no interaction between disease treatment and soil inoculum, **Table 2**). However, chrysanthemum growing with soil inocula conditioned by *Lolium perenne* and *Vicia sativa* had significantly higher aboveground biomass with *P. ultimum* than without *P. ultimum* addition (**Figure 1A**).

**Table 2:** Overall effects of identity and functional group of the conditioning plant species, and of *Pythium* addition on aboveground biomass, belowground biomass, root/shoot ratio, proportion of yellow leaves and leaf chlorogenic acid concentrations in chrysanthemum.

	df	Aboveground biomass	Belowground biomass	Root/shoot ratio	Yellowness	Chlorogenic acid
Species	36, 148	6.01***	5.18***	1.94**	1.62*	2.05**
*Pythium*	1, 148	2.83	23.83***	115.15***	0.13	5.87*
Species ×*Pythium*	36, 148	0.74	0.66	1.73*	1.40	1.47
Functional group	2, 34	14.30***	15.46***	5.89**	6.52**	2.71
*Pythium*	1, 182	2.97	25.57***	103.83***	0.01	5.20*
Functional group ×*Pythium*	2, 182	0.53	1.20	3.86*	0.02	1.57

*Data presented are degrees of freedom (df) and F-values from the linear mixed models and generalized linear mixed model (only used for yellowness). Asterisks indicate significant effects at ^∗∗∗^P < 0.001, ^∗∗^P < 0.01, ^∗^P < 0.05.*

Root biomass of chrysanthemum grown with inocula conditioned by *Centaurea jacea* and *Trifolium arvense* was significantly lower than that of plants grown in 100% sterile soil in the no-disease treatment (**Figure 1B**). Addition of 12 species-specific soil inocula resulted in lower chrysanthemum root biomass than no plant soil inoculum. Addition of *P. ultimum* caused a significant reduction in root biomass but the interaction between disease addition and soil inoculation was not significant (**Table 2**). Addition of *P. ultimum* in soil inoculated with *Agrostis stolonifera, Achillea millefolium, Tanacetum vulgare*, or *Tagetes minuta* soil resulted in a significant reduction in root biomass. Root/shoot ratios were significantly lower in soil with *P. ultimum* addition (Supplementary Figure 1) and the effects of *P. ultimum* addition differed between inocula resulting in a significant interaction between these two factors (**Table 2**). Grass species had neutral to positive plant–soil feedback effects on chrysanthemum, while forb and legume species had neutral to negative plant–soil feedback effects compared to sterile soil with or without *Pythium* addition (Supplementary Figure 2A). Most plant species had negative plant–soil feedback effects on chrysanthemum when compared with the no plant inoculum either with or without *Pythium* addition (Supplementary Figure 2B).

**FIGURE 2:**
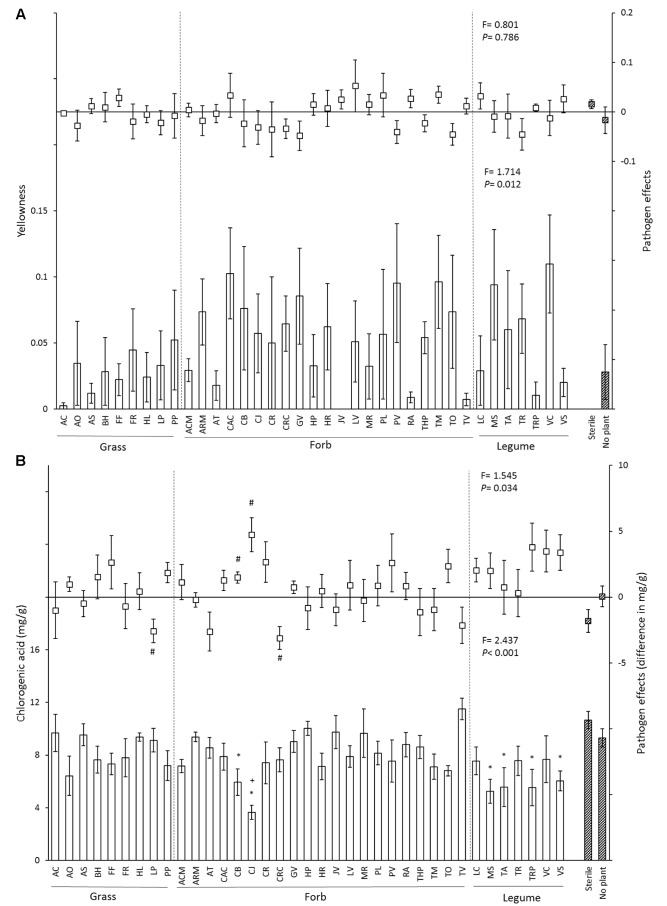
Effects of 37 species-specific soil inocula, no plant inoculum and sterile soil on chrysanthemum yellowness **(A)** and leaf chlorogenic acid concentration **(B)**. In each figure, bars represent the mean (±SE) of each soil inoculum in control soil, and squares represent the pathogen effect (value in *P. ultimum* soil – value in non-*Pythium* inoculated soil). Striped bars indicate controls. “^∗^” Represents significant difference from the sterile soil (*P* < 0.05). “+” Represents significant difference from the no plant soil inoculum (*P* < 0.05), “#” represents significantly different from zero (*P* < 0.05). Dashed lines separate soil inocula into different functional groups. Statistics presented in the lower part of each panel represent the effects of soil in control soil, and statistics presented in the upper part of each panel indicate the effects of soil inocula on the disease severity of chrysanthemum biomass.

The proportion of yellow leaves differed significantly between soil inocula (**Figure 2A** and **Table 2**). In the control treatment, leaf chlorogenic acid concentrations of plants growing in soils with *Capsella bursa-pastoris*, *Centaurea jacea*, *Medicago sativa*, *Trifolium arvense*, *Trifolium pratense*, and *Vicia sativa* inocula were significantly lower than in sterile soil, and leaf chlorogenic acid concentrations in soil conditioned by *Centaurea jacea* was significantly lower than no plant soil (**Figure 2B**). With *P. ultimum* inoculation, leaf chlorogenic acid concentrations of plants growing in soils with *Lolium perenne* and *Crepis capillaris* inocula were significantly lower than those in control treatment, while leaf chlorogenic acid concentrations of plants growing in soil conditioned by *Capsella bursa-pastoris*, *Centaurea jacea* were significantly higher than those growing in control soil (**Figure 2B**).

**FIGURE 3:**
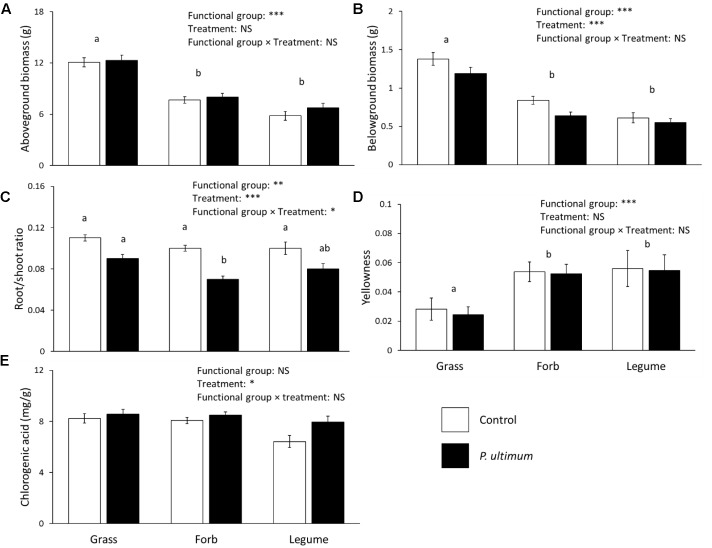
Effects of plant functional group and pathogen addition on chrysanthemum aboveground biomass **(A)**, belowground biomass **(B)**, root/shoot ratio **(C)**, proportion of yellow leaves **(D)**, and leaf chlorogenic acid concentration **(E)**. Data show means ± SE, with white bars representing control soil, and black bars representing the *P. ultimum* treatment. Different letters indicate significant differences between functional groups (*P* < 0.05). For root/shoot ratio, different letters above bars indicate significant differences (*P* < 0.05). Full statistics are listed in **Table 2**.

Both aboveground and belowground biomass of chrysanthemum differed significantly between functional groups of the conditioning plant species (**Figures 3A,B**). Addition of soil inocula created by grasses resulted in significantly higher above- and belowground biomass of chrysanthemum than addition of forb or legume inocula. The root/shoot ratio differed between functional groups of the conditioning plant species and disease treatment, there were interactions between functional groups and the disease treatment (**Figure 3C** and **Table 2**). Root/shoot ratios did not differ between grass, legume or forb inocula in control soil but in presence of *P. ultimum*, root/shoot ratios were significantly lower with forb than with grass inocula (**Figure 3C**).

The proportion of yellow leaves differed significantly between functional groups of the conditioning plant species (**Figure 3D**). *Pythium ultimum* inoculation did not significantly influence chrysanthemum yellowness. Addition of soil inocula created by grasses resulted in significantly lower chrysanthemum yellowness than addition of forb or legume inocula.

**FIGURE 4:**
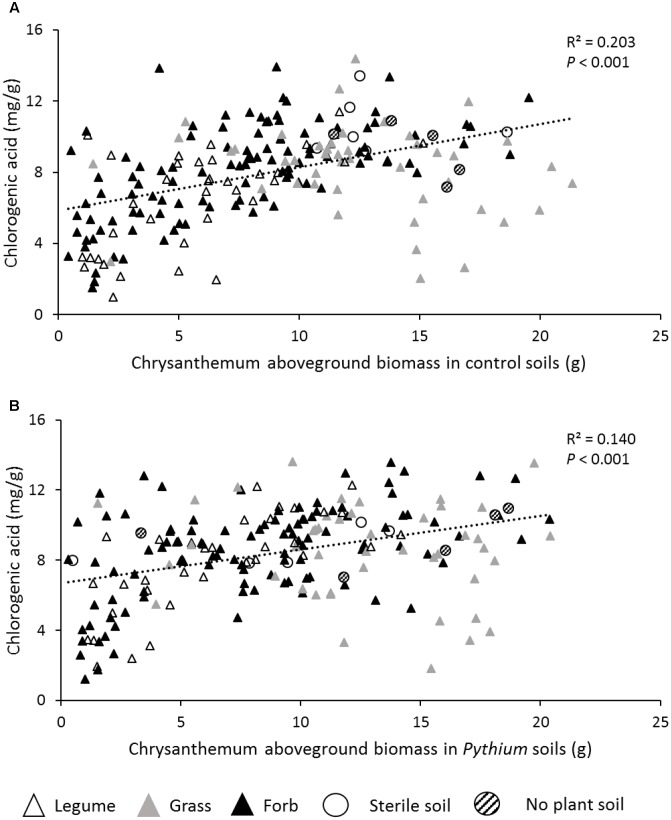
Relationships between chrysanthemum leaf chlorogenic acid concentration and aboveground biomass in control soil **(A)**, and *Pythium* added soils **(B)**. Black triangles represent forb inocula; Gray triangles represent grass inocula; White triangles represent legume inocula; White circles represent 100% sterile soil; Striped circles represent no plant soil.

The concentration of chlorogenic acid was significantly influenced by the identity of the plant species that was used to create the inoculum but did not differ between plant functional groups (**Figure 3E** and **Table 2**). The concentration of chlorogenic acid significantly increased in response to *P. ultimum* addition (**Figure 3E** and **Table 2**). Chlorogenic acid concentrations were positively related with chrysanthemum aboveground biomass in both the no-disease and disease treatments (**Figures 4A,B**).

**FIGURE 5:**
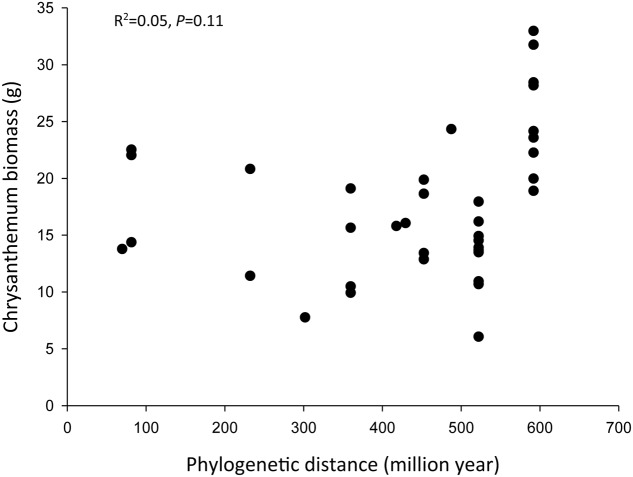
Effects of phylogenetic relationships on the chrysanthemum biomass (aboveground biomass + belowground biomass). The phylogenetic distance is the distance of conditioning plant species to chrysanthemum. The phylogenetic relationship is based on a backbone tree of the recent phylogenetic hypothesis of the Angiosperm Phylogeny Group (R20120829 for plants). We used the BLADJ algorithm of the Phylocom to get branch lengths scaled to time, based on clade ages according to Wikström et al. (2001).

There was no significant relationship between phylogenetic distance and the effect of the inoculum on chrysanthemum growth (*R*^2^= 0.05, *P* = 0.11) (**Figure 5**). Topology of the phylogenetic tree is given in Supplementary Figure 3.

## Discussion

Our study shows that the identity of the plant species that conditioned the soil had a large effect on the plant–soil feedback effects on chrysanthemum growth and that plant functional group is a strong determinant of plant–soil feedback effects. When quantifying plant–soil feedback effects relative to sterile soil, most legume and forb species had negative plant–soil feedback effects on chrysanthemum biomass. In contrast, grass species had neutral to positive feedback effects on chrysanthemum biomass, and this became more apparent when *Pythium* was added. Moreover, addition of grass inocula led to more biomass and less yellowness than addition of legume or forb inocula, and led to less strong *Pythium* effects than addition of forb inocula. Importantly, and contrary to our initial hypothesis, addition of soil inocula that were created by legumes did not result in positive effects on chrysanthemum growth and did not reduce disease severity.

Inoculation with eight of the 37 soil inocula we tested negatively influenced chrysanthemum biomass compared with growth on sterile soil. Interestingly, plants grown with *Lolium perenne* inoculum that were exposed to *P. ultimum* had higher aboveground biomass than plants without *P. ultimum*. *Lolium perenne* has a highly diverse soil microbial community (Wardle et al., 2003; Clayton et al., 2005), and this species has been reported to cause increases in the density of bacteria that produce biocontrol compounds, such as 2,4-diacetylphloroglucinol, pyrrolnitrin and hydrogen cyanide (Latz et al., 2015). Thus, chrysanthemum plants grown with *Lolium perenne* inoculum may have been primed by these rhizobacteria, so that later when exposed to *P. ultimum*, the plants could respond better and faster to pathogen invasion (Pieterse et al., 2014). Pathogen infection can also lead to higher root colonization of beneficial bacteria (Rudrappa et al., 2008; Liu et al., 2014). This may explain why the biomass of chrysanthemum grown with *Lolium perenne* inoculum was larger in presence of *P. ultimum* than without the pathogen.

Chrysanthemum grown in soil with grass inocula sustained higher above- and belowground biomass than plants grown with inocula conditioned by legumes or forbs. This is partially in line with our hypothesis that grass and legume inocula have a more positive influence on chrysanthemum growth than forb inocula. Other studies with the same and with different soils have shown that the composition of the microbial community of grass-conditioned soil differs distinctly from legume-conditioned soil (Chen et al., 2008; Kos et al., 2015). Several other studies have shown that grasses in particular increase the abundance of soil bacteria, such as *Bacillus*, *Pseudomonas* and *Actinomyces*, which can act as antagonists of soil pathogens (Latz et al., 2012, 2016; Chen et al., 2016). Moreover, grasses can also increase the abundance of AM-fungi (De Deyn et al., 2010). These mechanisms may explain the better effects of grass inocula relative to legume or forb inocula in our study. Grass inocula also sustained lower chrysanthemum yellowness than forb or legume inocula, and grass inocula overall increased plant growth and health more than legume or forb inocula. Steaming soil can kill both beneficial and pathogenic microbes in the soil, and this can lead to the rapid build-up of soil pathogens. Although grass-conditioned soil inocula did not enhance chrysanthemum growth more than that of plants grown in sterile soil, our study shows that it can provide other benefits to plants, e.g., higher resistance to pathogen infection. For example, in presence of *Pythium*, addition *Lolium perenne* inoculum, resulted in higher chrysanthemum aboveground biomass. Further studies concerning the microbial interactions between soil pathogen addition and species-specific soil inocula are needed to unravel the mechanism behind this.

Surprisingly and in contrast to our hypothesis, chrysanthemum performance was worse overall with legume inocula. Legumes are often used in crop rotation to increase nitrogen content of soils (Drinkwater et al., 1998). Since in our experiments chrysanthemum plants were heavily fertilized, a nitrogen-mediated benefit of legume soil is unlikely. In contrast, the negative influence of soil inocula conditioned by legumes on chrysanthemum growth could be explained by the negative effects of legumes on certain beneficial soil bacteria (Latz et al., 2012, 2015). Legumes produce steroid saponins that act as antifungal and antibacterial compounds (Mahato et al., 1982). Moreover, the rhizobia have similar colonization strategies to both legume and non-legume plants, however, rhizobia refine their strategy to symbiosis when interacting with legumes (Soto et al., 2006, 2009). Thus, for the non-leguminous plant chrysanthemum, rhizobia would act like pathogens, explaining the reduction of plant growth in soils conditioned by legumes. Addition of soil inocula created by forbs overall also significantly decreased chrysanthemum growth. Chrysanthemum root/shoot ratios indicated plant susceptibility to *Pythium*, as *Pythium* infection reduces the root system and leads to root rot (Agrios, 2005). There were no significant differences between chrysanthemum root/shoot ratios in grass, forb or legume inocula without *P. ultimum* addition. However, with *P. ultimum* addition, chrysanthemum root/shoot ratios of plants growing with in forb inocula decreased significantly more than that of plants growing with grass inocula, suggesting poor plant resistance to *P. ultimum* attack when grown with forb inocula. Forbs generally allocate less carbon to roots and have overall less microbial activity and abundance in roots than grasses (Warembourg et al., 2003; Chen et al., 2016). Hence, we speculate that the microbial community of soil inocula from forbs was smaller or less active or diverse than the microbial community of grasses. Whether this is true remains to be tested.

Plant–soil feedback effects can also be due to the modification of abiotic conditions (Ehrenfeld et al., 2005). However, in our study, we inoculated 90% homogenized sterile soil with 10% conditioned soil, and thus we minimized the heterogeneity of abiotic factors (Kardol et al., 2006). More importantly, in the feedback phase, plants received a high dose of Hoagland fertilizer following common practice in commercial chrysanthemum greenhouses. Thus it is highly unlikely that inocula-related differences in nutrient availability influenced the results in our study, and therefore we can assume that the different plant–soil feedback effects were due to differences in microbial communities. Nutrient-rich substrates are typically exploited by r-strategist species such as *P. ultimum*, and the suppression of *P. ultimum* can be difficult in soils with high nutrient levels (van Bruggen and Semenov, 2000). This may explain why the inocula were relative ineffective in suppressing *P. ultimum* infection.

Overall, the concentration of chlorogenic acid in chrysanthemum leaves differed significantly between the inocula. However, although the concentration of leaf chlorogenic acid was positively related with aboveground plant biomass, and grass inocula sustained significantly higher chrysanthemum aboveground biomass compared to forb inocula or legume inocula, the concentration of chlorogenic acid in grass inocula did not differ from those in legume inocula or forb inocula. The concentration of leaf chlorogenic acid was found to be positively correlated with plant carbon assimilation rates in sorghum (Turner et al., 2016). In our study, the levels of aboveground chlorogenic acid also increased with pathogen attack belowground compared to uninfected plants. Soil pathogens can increase aboveground plant defense even in absence of aboveground plant antagonists (Bezemer and van Dam, 2005). In chrysanthemum, chlorogenic acid is related to resistance against thrips (Leiss et al., 2009, 2011), as well as to other herbivores, such as leafminers and spider mites (Kos et al., 2014). Our work therefore suggests that soil inoculation but also the presence of soil pathogens can influence the resistance of chrysanthemum against aboveground herbivorous pests and that plant–soil feedback effects may influence pest severity and biocontrol in chrysanthemum cultivations.

In contrast to our hypothesis, the plant–soil feedback effect of species closely related to chrysanthemum was not more severe than that of distantly related species. It may be possible that beyond a certain threshold phylogenetic distance, effects do become apparent, as shown by the grass clade, which is the most distantly related one. To prove this, future studies should select species across large phylogenetic scales to test their plant–soil feedback effects. Our result is in line with an increasing number of studies with wild plant species showing that phylogenetic distance is a poor predictor of plant–soil feedback effects (Pavoine et al., 2013; Kelly et al., 2014; Mehrabi and Tuck, 2015; Mehrabi et al., 2015). Thus, although our study demonstrated species specific plant–soil feedback effects, these patterns may not correspond to mechanisms like shared pathogens or symbionts. Moreover, there is a growing awareness that the phylogenetic distance is a weak predictor of the dissimilarity of plant functional traits (Mouquet et al., 2012; Pavoine et al., 2013; Kelly et al., 2014). If for example, traits responsible for resource use or host susceptibility to natural enemies are not conserved, the plant species will influence or respond to the soil in a very different way even though they are closely related (Mehrabi and Tuck, 2015). Several recent studies have shown that PSF effects can be predicted from life history forms or plant traits such as root thickness or density or plant growth rate (Baxendale et al., 2014; Cortois et al., 2016; De Deyn, 2017). Therefor, plant traits instead of phylogenetic distance could be a good predictor of plant soil feedback effects.

## Conclusion

In summary, we demonstrate that plant species through changes in the soil can influence the growth, disease susceptibility and the concentration of aboveground defense compounds of cultivated crop species, all in a species-specific manner. Our results further show clearly that these plant–soil feedback effects depend on plant functional groups of the species where the inocula are created from, with the highest chrysanthemum performance in soil with grass inocula. Our study with a cultivated plant species highlights that species-specific plant–soil feedback effects can also play an important role in deciphering interactions between plants and pathogens or herbivorous insects in horticulture. Disentangling the mechanisms of enhanced plant performance, and evaluating the consequences for plant yield in a real horticultural setting may allow us to implement the concept of plant–soil feedbacks in current greenhouse horticulture.

## Author Contributions

H-KM, AvdW, and TMB conceived the ideas and designed methodology; H-KM, CR, and TMB collected the data; H-KM, AP, and TMB analyzed the data; H-KM, AP, and TMB led the writing of the manuscript. All authors contributed critically to the drafts and gave final approval for publication.

## Conflict of Interest Statement

The authors declare that the research was conducted in the absence of any commercial or financial relationships that could be construed as a potential conflict of interest.
