# Supplementary material for: Plant–Soil Feedback Effects on Growth, Defense and Susceptibility to a Soil-Borne Disease in a Cut Flower Crop: Species and Functional Group Effects
Source: Front Plant Sci. 2017 Dec 19;8:2127. doi: 10.3389/fpls.2017.02127 (PMC5742127; doi:10.3389/fpls.2017.02127)
Supplement: Supplementary file 1 [file Image_1.pdf]

Supplementary Material

Plant-soil feedback effects on growth, defense and susceptibility to a soil-borne disease in cut flower crop: species and functional group effects

Hai-kun Ma[*], Ana Pineda, Andre W.G. van der Wurff, Ciska Raaijmakers, T. Martijn Bezemer

*** Correspondence:** Corresponding Author: H.Ma@nioo.knaw.nl

Supplementary Figures

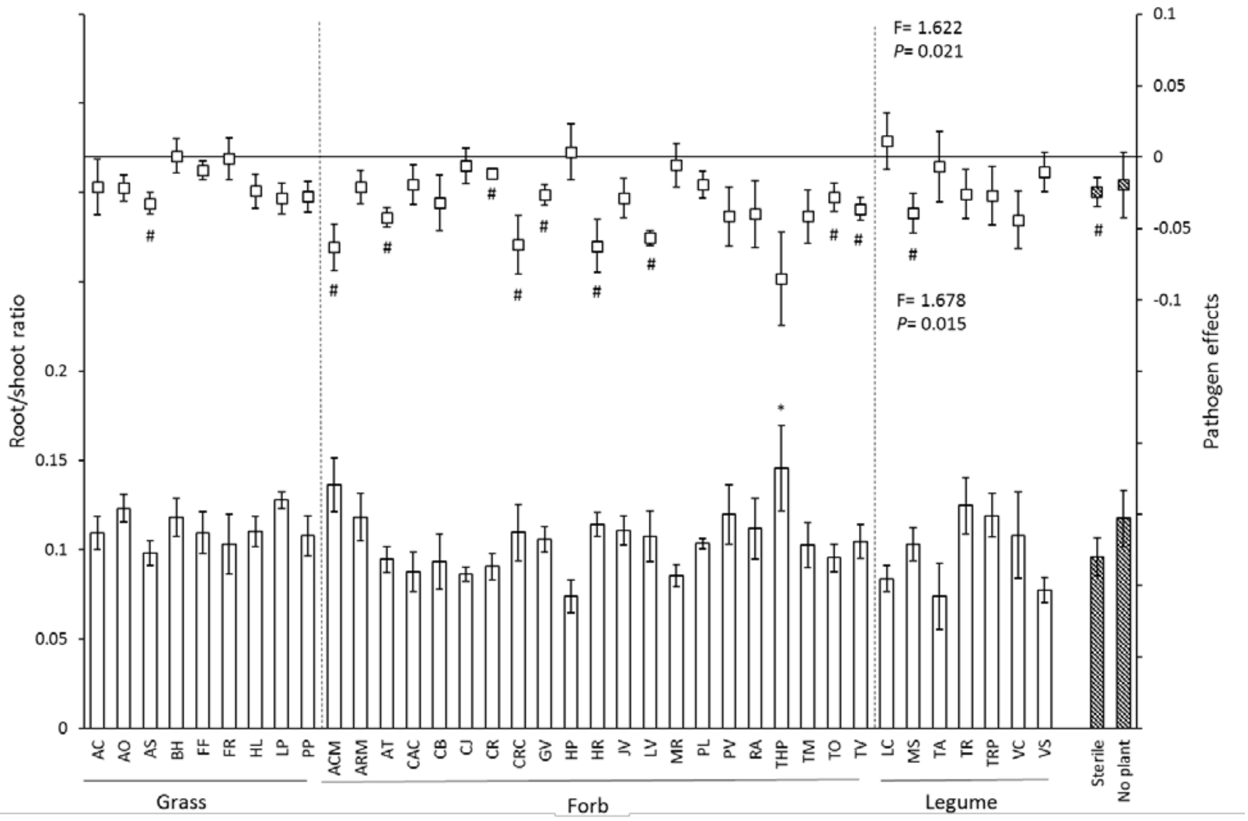

Supplementary Figure 1. Effects of 37 species-specific soil inocula, no plant inocula and sterile soil on chrysanthemum root/shoot ratio. In each figure, bars represent chrysanthemum biomass (mean ± SE) of soil inocula in control soil, and squares represent the pathogen effect on plant biomass (root/shoot ratio in *P. ultimum* soil – root/shoot ratio in non-*Pythium* soil). Stripe bars indicate controls. "*" represents significant difference from the sterile soil ($P < 0.05$). "+" represents significant difference from the no plant soil inocula ($P < 0.05$), "#" represents significantly different from zero ($P < 0.05$). Dashed lines separate soil inocula into

different functional groups. Species abbreviations are given in Table 1. Statistics presented in the lower part of each pannel represent the effects of soil on chrysanthemum root/shoot ratio in control soil, and statistics presented in the upper part of each pannel indicate the effects of soil inocula on the disease severity of chrysanthemum root/shoot ratio.
