# Supplementary material for: Plant–Soil Feedback Effects on Growth, Defense and Susceptibility to a Soil-Borne Disease in a Cut Flower Crop: Species and Functional Group Effects
Source: Front Plant Sci. 2017 Dec 19;8:2127. doi: 10.3389/fpls.2017.02127 (PMC5742127; doi:10.3389/fpls.2017.02127)
Supplement: Supplementary file 2 [file Image_2.PDF]

Supplementary Material

Plant-soil feedback effects on growth, defense and susceptibility to a soil-borne disease in cut flower crop: species and functional group effects

Hai-kun Ma*, Ana Pineda, Andre W.G. van der Wurff, Ciska Raaijmakers, T. Martijn Bezemer

*** Correspondence:** Corresponding Author: H.Ma@nioo.knaw.nl

Supplementary Figures

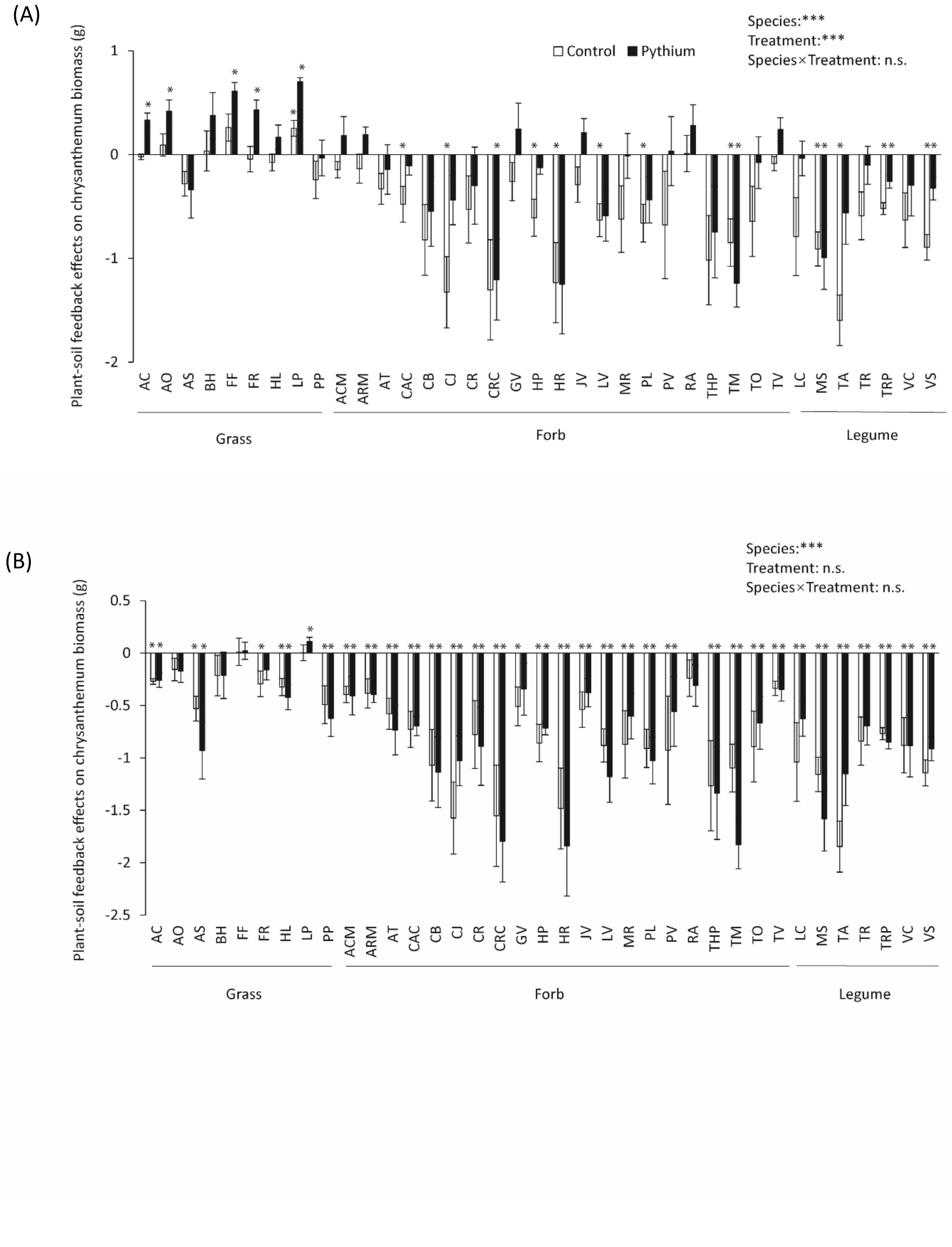

Supplementary Figure 2. Plant-soil feedback effects of 37 plant species on chrysanthemum biomass (mean ± SE). Chrysanthemum biomass calculated as the sum of aboveground biomass and belowground biomass. (A) The plant–soil feedback effect of soil conditioning by a species on chrysanthemum biomass was calculated as the natural logarithm of chrysanthemum biomass on soil conditioned by that species minus the natural logarithm of average chrysanthemum biomass on 100% sterile soil. (B) The plant–soil feedback effect of soil conditioning by a species on chrysanthemum biomass was calculated as the natural

logarithm of chrysanthemum biomass on soil conditioned by that species minus the natural logarithm of average chrysanthemum biomass on no plant soil. White bars indicate control treatment; black bars indicate *P. ultimum* treatment. “*” above each bar indicates significant difference from zero (P < 0.05), suggesting significant difference from sterile soil. Overall effects of conditioning plant species, disease treatment, and the interaction between the two from a two-way ANOVA are present on the graph. “*” indicates significant effects. “n.s.” indicates no significant effects. Species abbreviations are given in Table 1.
