# Supplementary material for: Plant–Soil Feedback Effects on Growth, Defense and Susceptibility to a Soil-Borne Disease in a Cut Flower Crop: Species and Functional Group Effects
Source: Front Plant Sci. 2017 Dec 19;8:2127. doi: 10.3389/fpls.2017.02127 (PMC5742127; doi:10.3389/fpls.2017.02127)
Supplement: Supplementary file 3 [file Image_3.pdf]

Supplementary Material

Plant-soil feedback effects on growth, defense and susceptibility to a soil-borne disease in cut flower crop: species and functional group effects

Hai-kun Ma*, Ana Pineda, Andre W.G. van der Wurff, Ciska Raaijmakers, T. Martijn Bezemer

*** Correspondence:** Corresponding Author: H.Ma@nioo.knaw.nl

Supplementary Figures

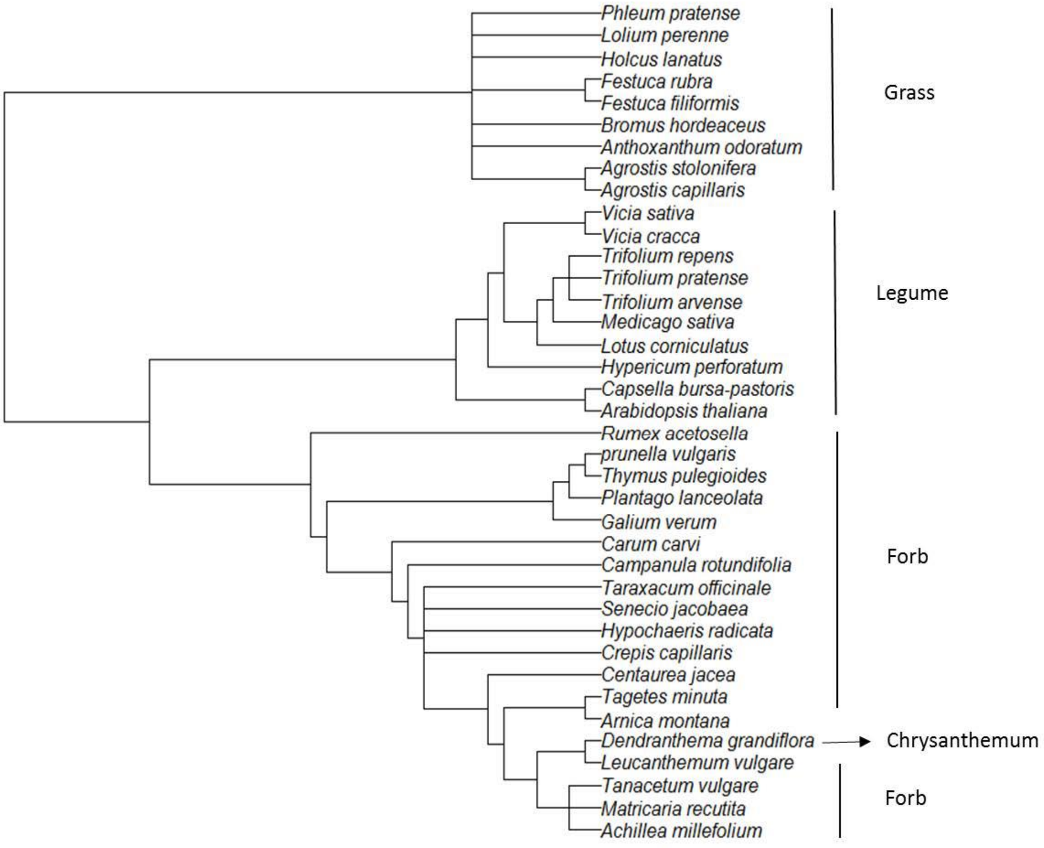

Supplementary Figure 3. Phylogenetic relationships between conditioned plant species and chrysanthemum. Topology from Phylomatic program (Webb & Donoghue 2005).
